# HIV infection and HERV expression: a review

**DOI:** 10.1186/1742-4690-9-6

**Published:** 2012-01-16

**Authors:** Antoinette C van der Kuyl

**Affiliations:** 1 Laboratory of Experimental Virology, Department of Medical Microbiology, Center for Infection and Immunity Amsterdam (CINIMA), Academic Medical Center of the University of Amsterdam, Meibergdreef 15, 1105 AZ Amsterdam, The Netherlands

## Abstract

The human genome contains multiple copies of retrovirus genomes known as endogenous retroviruses (ERVs) that have entered the germ-line at some point in evolution. Several of these proviruses have retained (partial) coding capacity, so that a number of viral proteins or even virus particles are expressed under various conditions. Human ERVs (HERVs) belong to the beta-, gamma-, or spuma- retrovirus groups. Endogenous delta- and lenti- viruses are notably absent in humans, although endogenous lentivirus genomes have been found in lower primates. Exogenous retroviruses that currently form a health threat to humans intriguingly belong to those absent groups. The best studied of the two infectious human retroviruses is the lentivirus human immunodeficiency virus (HIV) which has an overwhelming influence on its host by infecting cells of the immune system. One HIV-induced change is the induction of HERV transcription, often leading to induced HERV protein expression. This review will discuss the potential HIV-HERV interactions.

Several studies have suggested that HERV proteins are unlikely to complement defective HIV virions, nor is HIV able to package HERV transcripts, probably due to low levels of sequence similarity. It is unclear whether the expression of HERVs has a negative, neutral, or positive influence on HIV-AIDS disease progression. A positive effect was recently reported by the specific expression of HERVs in chronically HIV-infected patients, which results in the presentation of HERV-derived peptides to CD8^+ ^T-cells. These cytotoxic T-cells were not tolerant to HERV peptides, as would be expected for self-antigens, and consequently lysed the HIV-infected, HERV-presenting cells. This novel mechanism could control HIV replication and result in a low plasma viral load. The possibility of developing a vaccination strategy based on these HERV peptides will be discussed.

## Review

Retroviruses are unique among the viridae by inserting their genome into the host cellular DNA as an essential step in the viral replication cycle. Some older members of the retrovirus family have found their way into the vertebrate germ line while current members seem to remain exogenous. Vertebrate genomes contain substantial amounts of retroviral sequences in various states of inactivation since their integration (for a review on the discovery, see [1]). Integrated endogenous retrovirus (ERV) genomes commonly contain mutations, deletions, or are even reduced to single long terminal repeat (LTR) elements by homologous recombination between the two LTR's. More recent integrations usually have retained at least partial coding capacity. Some integrated ERV elements have been adopted and are used to the hosts' advantage, either as a novel regulatory sequence, a novel protein, or as a means to protect against new retrovirus infections (reviewed by [2]). This latter mechanism is called superinfection resistance (SIR), and works best against closely related retroviruses by simple mechanisms such as receptor occupancy (reviewed by [3]).

**Figure 1:**
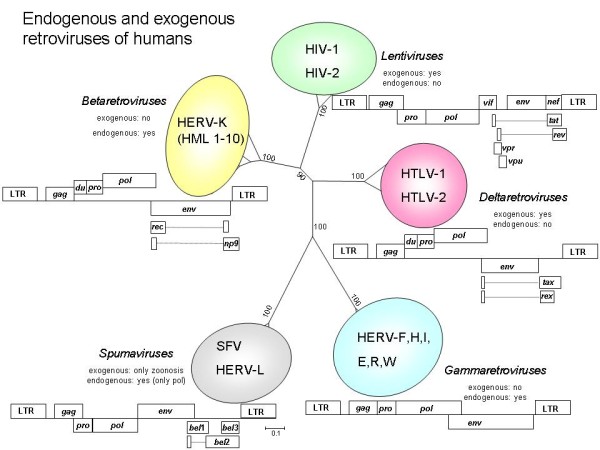
**Phylogenetic tree of human retroviruses**. A 183 translated amino acid fragment surrounding the YXDD motif in the *pol *gene shows the relationship between endogenous and exogenous retroviruses of humans. Sequences were retrieved from the GenBank database [83], translated and aligned using BioEdit version 7.0 [84]. A phylogenetic tree with 500 bootstrap replicates was constructed with the neighbour-joining method based upon a distance matrix generated with the Poisson model for amino acid substitutions while assuming uniform rates among sites, as implemented in MEGA 5.0 [85]. Schematic genome organizations for different classes of retroviruses are shown. Drawings are not to scale. Additional reading frames may exist in other strains (e.g. non-primate lentiviruses encode a dUTPase). Abbreviations: HERV = human endogenous retrovirus; HIV = human immunodeficiency virus; HTLV = human T-cell lymphotropic virus; SFV = simian foamy virus. Accession numbers: AF074086 (HERV-K HML-2); NC_001436 (HTLV-1); M10060 (HTLV-2); K03455 (HIV-1); M15390 (HIV-2); U04327 (SFV); NT_029419 (from which a HERV-E Pol sequence was retrieved). Analysed but not shown: AF033807 (MMTV, a betaretrovirus); J01998 (MuLV, a gammaretrovirus); M12349 (simian Mason-Pfizer monkey virus, a deltaretrovirus); DQ399707 (XMRV, a gammaretrovirus), and the horse endogenous betaretrovirus EqERV-beta 1 [86]. LTR = long terminal repeat, consisting of the U3, R and U5 regions in the integrated provirus, *gag *= group-specific-antigen, *du *= dUTPase, *pro *= protease, *pol *= polymerase (reverse transcriptase and integrase), *env *= envelope, *bel 1-3 (bel 1 *is also known as *tas; *the *bel 2 *reading frame overlaps with another one named *bet)*, *tax*, *rex*, *tat*, *rev*, *vpu*, *vif*, *nef *and *vpr *encode small additional proteins. The HERV-K Rec protein is also known as K-Rev. HERV-K *rec *is found in HERV-K type II proviruses, while *np9 *is encoded by HERV-K type I proviruses [87]. In spumavirus, either *gag-pro *or *pro-pol *are encoded in the same translational reading frame.

Around 8% of the human genome is of retroviral origin, which includes proviruses that belong to beta-, gamma-, and spuma- retroviruses (Figure 1). Human endogenous retroviruses (HERVs) are historically classified by the tRNA specificity of their primer binding site (PBS), which can be confusing as unrelated species may share the same tRNA primer for reverse transcription [4]. Many HERV elements may have lost the ability to transfer, but several retain the capability to be transcribed and translated under certain conditions, including embryonic development and disease [2]. The most recent and widespread human integrations belong to retroviruses with homology to mouse mammary tumour virus (MMTV, a betaretrovirus) and are known as the HERV-K (HML-2) (human MMTV-like) family (for a recent review, see [5]). Full-length proviral genomes of HML-2 family members are present, but these are not replication competent, even the HERV-K113 element that retains full coding capacity [6]. The human germ line tumour cell Tera-1 even produces (non-infectious) retrovirus particles containing HML-2 RNA, but the assembly of these particles was found to depend on trans-acting viral proteins and RNA genomes derived from a mosaic of HML-2 proviral genomes [7]. The human mammary carcinoma cell line T74-D was found to release virions with B-type morphology that also contain retroviral transcripts originating from different loci [8]. Infectious HML-2 viruses have now been reconstructed in the laboratory to delineate their characteristics [9,10].

Humans are currently threatened by only two exogenous retrovirus species: human T-cell lymphotropic virus (HTLV, a deltaretrovirus) and human immunodeficiency virus (HIV, a lentivirus). A third putative human retrovirus, xenotropic murine leukaemia virus-related virus (XMRV, a gammaretrovirus) has recently been discarded as a contaminant [11,12]. No sequences with homology to delta- or lenti- viruses have been found in the human genome, although endogenous lentivirus genomes were discovered in basal primates [13,14]. Infection with the spumavirus simian foamy virus (SFV) occurs occasionally in persons exposed to non-human primates, but the virus does not seem to spread among humans [15-17]. The only described human endogenous retrovirus element with (distant) homology to foamy viruses is HERV-L [18].

In this review, the effect of infection with an exogenous retrovirus (HIV) on the resident endogenous virus population will be summarized with regard to viral genome expression, possible complementation, and immune response.

## Complementation of HERV and HIV proteins

All retroviruses have a similar genetic make-up, whereby long terminal repeat (LTR) regulatory sequences flank the reading frames for Gag, Pol, and Env proteins. Gag and Pol are processed by the viral protease into smaller, functional proteins, while a cellular protease processes Env. The Pol polyprotein is cleaved into the protease (PR), reverse transcriptase (RT), and integrase (INT) enzymes. Homologous proteins encoded by one retrovirus could theoretically perform comparable functions *in trans *for another member of the family, and could thus complement a (defective) virus. Examples of possible complementation between HERV and HIV proteins will be discussed next.

A first example of a potential supplementary function is the dUTP pyrophophatase (dUTPase) enzyme. In betaretroviruses, a dUTPase domain is present at the N-terminal part of the protease. In non-primate exogenous lentiviruses, the dUTPase domain is located between the RNase H domain and the *integrase *coding region of the *pol *gene. Such dUTPase activity is essential for retrovirus replication by reducing the levels of dUTP during cDNA synthesis, thereby minimizing the incorporation of dUTP into the nascent DNA by the reverse transcriptase. In contrast to all other lentiviruses, exogenous primate lentiviruses (simian immunodefiency virus, SIV, and HIV) lack a dUTPase and depend upon the cellular uracil DNA-glycosylase UNG2 to provide this function [19]. Some researchers have suggested that HIV-1 can efficiently replicate without UNG2 [20], because it tolerates heavily uracilated DNA. Alternatively, the dUTPase encoded by active HERV-K proviruses could supply this function [21]. Interestingly, the endogenous lentivirus elements carried by lemurs do contain a *dUTPase *gene [13,14], suggesting that this function was specifically lost in the extant SIV lineage.

When protease inhibitors specifically designed to inhibit the HIV-PR enzyme were included in the drug regimen offered to HIV-infected patients, there was some concern as to whether endogenous proteases, likely resistant to the anti-HIV drugs because of remote amino acid similarity, would be able to trans-complement the targeted enzyme. HERV-K10 protease is indeed highly resistant to HIV-PR inhibitors and able to process the HIV gag matrix-capsid protein at the authentic cleavage site [22]. However, when a HERV-K PR was incorporated into virions of a PR-defective HIV-1 strain, no complementation was achieved [23]. The Gag-Pol precursors were found to be processed at unnatural sites, rendering the produced virion particles non-infectious. When HERV-K PR was incorporated into wild type (wt) HIV-1 virions with the intention to decrease their infectivity, no deleterious effect was seen, and only small fractions of aberrantly cleaved proteins were detected [23].

HERV-K10 INT has been reported to be able to complement an INT-defective HIV-1 strain, although infectivity was severely reduced to 3.7% of wt HIV-1 [24]. It is nevertheless remarkable that HERV-K10 INT was able to recognize the dissimilar HIV-1 LTR sequences to achieve *in vitro *integration levels of 10-30% of HIV-1 INT.

Retroviral *envelope *proteins are quite versatile and can often be used to package dissimilar viral genomes (pseudotyping). A few HERV *envelope *genes, such as the gammaretroviral HERV-W derived *envelope *protein, syncytin, that plays a role in the attachment of the human placenta, are expressed *in vivo *[25,26]. HERV-W Env can still function as a viral envelope protein, as infectious particles were generated when it was used to complement an *env*-defective HIV-1 strain [27]. The HERV-W pseudotyped particles were infectious for CD4-negative cells, suggesting a mechanism by which HIV-1 can expand its cellular tropism during natural infection. Interestingly, *env*-deficient HIV-1 cannot be complemented by the Env protein of murine leukaemia virus (MuLV), also a gammaretrovirus [26,27]. In contrast, the generation of pseudotypes between HIV-1 and endogenous xenotropic MuLVs was proposed earlier as an explanation for the increased tropism of HIV-1 particles harvested from a cell line that had been passaged in immunosuppressed mice [28].

Some classes of retroviruses, termed complex retroviruses, encode several accessory proteins that simple retroviruses lack. For instance, HIV-1 encodes at least 6 additional proteins, betaretroviruses generally only one, and gammaretroviruses commonly none. An essential additional protein that assists in transporting unspliced and incompletely spliced viral mRNAs from the nucleus to the cytoplasm is named Rev in HIV-1 and Rex in HTLV. The trans-acting Rev protein binds to a viral RNA structure in Env-encoding sequences that is denoted the Rev responsive element (RRE). A homolog of Rev/Rex is encoded by HERV-K, where it is termed K-Rev or Rec [29]. To some extent, Rev/Rex proteins from complex retroviruses can complement each other, e.g. the Rex protein of HTLV-1 can functionally replace the HIV-1 Rev protein [30]. However, Rev/Rex complementation studies are not always straightforward because results often depend upon the experimental system used (see e.g. [31] and references therein). HIV-1 Rev can bind the HERV-K RRE, but the reverse is not true as K-Rev does not interact with the HIV-1 RRE [29]. Thus, although no actual complementation studies between HIV-Rev and K-Rev have been performed, it is unlikely that K-Rev can complement HIV-1 Rev because RRE binding is essential for protein function. Patients harbouring viral genomes with a *rev *defect or attenuating mutations have been described [32-34]. The disease phenotype in these patients is generally mild, suggesting that Rev defects *in vivo *are indeed not (completely) resolved by endogenous K-Rev.

## No HERV RNA in HIV-1 virions?

Retrovirus particles contain, apart from two copies of the viral RNA genome, spliced viral RNA's, several tRNAs, and varying amounts of cellular mRNAs that are co-packaged in a concentration dependent manner [35-37]. Some of the cellular RNAs, such as those coding for ribosomal proteins, are preferentially packaged in HIV virions [35]. If HERV RNA would be co-packaged into HIV-1 virions, this could result in effective mobilization of these elements. This was investigated in several packaging cell lines used in retroviral vector systems. Zeilfelder *et al*. showed that HIV-1 derived vectors systems incorporate very little HERV transcripts in particles in contrast to murine leukaemia virus (MLV) based vectors [38]. Although the system used by Zeilfelder *et al*. is highly artificial, it nevertheless suggests that HIV-1 does not readily encapsidate HERV transcripts or genomes. An explanation could be that HIV-1 Gag does not recognize the RNA packaging signals carried by the non-lentiviral HERVs, while MLV Gag is able to bind to the more related HERV sequences.

## Increase of HERV viral RNA after HIV-1 infection

*In vitro *HIV-1 infection of either CD4 expressing T- cell lines or stimulated peripheral blood mononuclear cells (PBMC's) upregulates the production of HERV-K RNA and proteins compared to non-infected cells [39]. Analysis of blood plasma samples from HIV-1 infected patients also showed a significant increase of HERV-K RNA expression [40,41], with titers as high as 10^6^-10^10 ^copies/ml [42]. In > 95% of samples HERV-K *pol *RNA was found compared to only 5-8% of the control samples, which included hepatitis C virus (HCV) infected patients and healthy individuals [40]. In contrast, HERV-H RNA, which is upregulated in rheumatoid arthritis patients, was not detected in HIV-1 infected blood plasma [40]. The majority of the HERV transcripts in HIV-1 infected samples belonged to the HERV-K (HML-2) family and a minority to the HERV-K (HML-3) cluster. These HERV-K transcripts were derived from several genomic loci, but transcripts from the HML-2 type-2 provirus located at chromosome 4q35 predominated [42]. HML-2 type 1 and type 2 proviruses differ from each other by a 292 nt deletion in the *env *gene that disables the expression of functional protein in type 1. Further analysis suggested that the observed transcripts likely represent full-length viral RNAs that are protected from degradation by Gag protein [40]. Indeed, immature virus particles that reacted only with anti-Gag Ig were observed using electron microscopy in a follow-up study, although mature viral particles reacting with anti-HERV-K Env antibodies and possessing condensed cores and symmetrically distributed spikes were also occasionally present in the blood plasma of HIV-1 infected patients [42]. HERV-K recombinant genomes were detected in blood plasma from some HIV-1 infected patients but not in breast cancer patients. Furthermore, genetic variation was observed in *env *(primarily synonymous substitutions indicative of purifying selection), with preservation of glycosylation sites together suggestive of replication through reverse transcription [42].

Laderoute *et al*. focussed on a HERV-K102 member of the HML-2 family that is upregulated during HIV-1 infection [43]. They reported that ~ 76% of blood plasma samples of HIV-1 infected patients test positive for particle associated HERV-K102 *pol *transcripts, versus ~ 3% of healthy controls. However, ~ 79% and ~ 62% of individuals with hepatitis B virus (HBV), hepatitis C virus (HCV) or herpes viremia also showed increased particle associated HERV-K102 *pol *transcript expression [43], suggesting a more general mechanism of HERV-K upregulation due to virus infection and immune stimulation. HERV-K upregulation was independent of ethnicity as it was seen both in individuals of European and African descent [43].

In contrast, high throughput sequencing of transcripts from HIV-1 infected versus uninfected cells did not demonstrate a significant increase in HERV expression upon HIV-1 infection [44]. Possibly, the genetically highly aberrant SupT1 T-cell line used in these experiments can account for this unexpected result. Either HERV expression could already be at peak level before HIV infection of these cells, or specific HERV integrations could simply be absent. Alternatively, the vesicular stomatitis virus (VSV)-G envelope protein pseudo-typed HIV particles used could possibly explain the lack of HERV induction, as Contreras-Galindo *et al*. [39] used complete HIV-1 viral particles in their infection assays. Besides, virus infection conditions differed significantly between the experiments.

A significant upregulation of HERV-W and HERV-K RNA was detected in brain tissue from patients with AIDS dementia, whereas HERV-E expression was decreased [45]. The changes in HERV expression were associated with monocyte differentiation and macrophage activation.

## HERV expression during antiretroviral therapy

As mentioned before, HERV-K RNA expression is increased in a dose-dependent manner by infection of peripheral blood mononuclear cells (PBMC's) and various cell lines with HIV-1 viral particles [39]. *In vivo*, it has not been examined whether HERV-K RNA levels increase during acute or chronic HIV-1 infection. Highly-active antiretroviral therapy (HAART) against HIV decreases the HIV plasma viral load (pVL) to < 50 copies/ml in most patients. Quantifying the HERV-K RNA load in patients receiving HAART confirmed that the HERV-K load is linked to the HIV-1 pVL. Patients that showed a good suppression of HIV-1 replication had a HERV-K load that was around two orders of magnitude lower than the average HERV-K load in patients with less efficient HIV-1 suppression [46]. HERV-K102 particle-associated nucleic acid could not be measured in five patients on antiretroviral therapy (four of them with good control of the HIV-1 pVL) in another study, in contrast to patients not receiving antiretroviral therapy [43].

Longitudinal analysis of patients receiving HAART demonstrated the long-term suppression of the HERV-K pVL to < 5000 copies/ml when the HIV-1 pVL remained below 50 copies/ml [47]. When patients failed HAART, the HERV-K pVL increased rapidly, frequently preceding the HIV-1 pVL rebound [47]. It is unclear whether HERV-K is (partially) inhibited by the antiretroviral drugs, or whether the loss of activation by HIV-1, or possibly another mechanism, is responsible for the reduction in HERV-K load. Protease inhibitors targeted at HIV are not active against the HERV-K enzyme [22], but other classes of antiretroviral drugs, in particular the nucleotide analogues that target the RT enzyme, might be. It is, however, not likely that the HERV-encoded reverse transcriptases are appreciably involved in the increase in HERV load, as copy numbers of both intact *RT *genes encoding functional proteins as well as intact primer binding sites (PBS) and other motifs needed to initiate reverse transcription, such as the primer activation domain (PAS) in HIV, are likely to be low. Also, RT enzymes prefer certain tRNA molecules for initiation of replication and e.g. HIV-1 RT has decreased activity when using other tRNA/PBS combinations [48,49]. This preference suggests that RT activity on non-self templates is probably greatly reduced, disabling the reverse transcription of non-family members.

Interestingly, the non-nucleoside RT inhibitors nevirapine and efavirenz can efficiently inhibit endogenous RT activity that is detectable in many human cell lines and leukaemic cells, thereby attenuating the malignant phenotype of the cells [50,51]. Nevirapine was not active against LINE-1 retroposon-encoded RT, whereas several nucleoside analogue RT inhibitors did inhibit LINE-1 RT, albeit with varying degrees of efficacy [52].

## Mechanisms of HERV activation by HIV infection

It is not clear what the precise mechanism of HERV induction by HIV infection is. HERV expression is suppressed in the germ-line and during the first steps of embryogenesis, while it is controlled during cell development (reviewed in [53]). Increased HERV expression is often seen during carcinogenesis and in autoimmune diseases such as multiple sclerosis [2], but it is unclear whether the increased expression is a cause or consequence of the disease. HERVs including the HERV-K (HML-2) family [54] and HIV-1 proviruses [55] are normally transcriptionally silenced by CpG methylation that is subsequently guarded by DNA methyltransferase 1 (DNMT1) (reviewed in [53]). Loss of methylation results in activation of proviruses, either HERV or HIV, but it has not been investigated whether HIV actively influences proviral methylation levels. HERV expression in HIV-infected cells could be enhanced if methylation levels of proviral DNA are reduced by HIV. Other investigators proposed a more complex mechanism for the induction of HIV-1 latency that employs transcriptional interference [56,57]. Several mechanisms may cooperate to restrict HIV provirus activation [58]. It is not clear whether changes in the pool of available transcription factors modulates HERV expression. Many HERV LTR promoters are probably not fully functional, e.g. not able to bind these transcription factors, and thus do no longer require such a repressive mechanism. Additionally, the HIV-1 proviral integration site location might also select for viral latency, virus induction or stable expression [59]. Generally, HIV-1 favours integration into transcriptionally active genes, usually in introns that are part of local transcription hotspots, without apparent directional bias [60]. In contrast, HERV proviruses are generally found outside genes, and commonly in the reverse orientation compared to the nearby cellular gene. HERV integrations seen in contemporary genomes are the result of stringent evolutionary selection, whereby HERV's in the sense orientation (facilitates transcription with nearby genes) or within cellular genes were probably negatively selected for [60]. In other words, observed HERV integrations do probably not faithfully reflect HERV target site preferences. Anyhow, the consequence is that HIV-1 proviruses are rarely found in the proximity of HERV integrations [59,60], ruling out that there is a direct positional effect of HIV-1 transcription upon HERV activation.

Specific upregulation of endogenous proviruses by HIV proteins or cellular proteins upregulated by HIV is another possibility. It has been suggested that the HIV-1 Tat protein is able to directly activate the HERV-K (HML-2) LTR [47]. The HTLV-I encoded transactivator protein Tax is likewise able to activate HERV LTRs, mainly of HERV-W and HERV-H [61]. HERV-K (HML-2) RNA is not, or only weakly, upregulated by common stimulation of donor PBMCs with phytohaemagglutinin, gamma irradiation or 5-azacytidine [62], suggesting that HERV upregulation requires undefined, yet specific conditions.

Another indirect mechanism of HERV upregulation by HIV-1 infection could be through opportunistic viral infections. HIV infection deregulates and eventually destroys the immune system. Loss of immune control facilitates the replication of diverse opportunistic pathogens. Some of these pathogens, e.g. herpesviruses, are virtually ubiquitous in the human population and remain latently present during the lifetime of the host. Herpesviruses are reactivated from latency during chronic HIV infection [63]. Herpes simplex virus type 1 (HSV-1) can specifically activate the HERV-W LTR, and induce expression of HERV-W Gag and Env proteins in cell lines [64-66]. Epstein-Barr virus (EBV), that has a prevalence rate of around 75% in humans, induces transcription of the *env *gene of HERV-K18 [67], and so do human herpesvirus 6A [68] and 6B [69]. Most likely, the other five human herpesviruses are also able to activate HERV transcription. HHV-6 and human herpesvirus 7 (HHV-7), that have infection rates of 100% each in the human population, infect, similar to HIV, CD4^+ ^T-cells, and could thus (partly) be responsible for HERV induction seen in PBMC's after HIV-1 infection. EBV commonly infects B-cells, while cytomegalovirus (CMV), with a prevalence rate of around 50%, infects monocytes and macrophages, and both could likewise contribute to the observed rise in PBMC HERV expression. In addition, infection by influenza A virus enhanced HERV-W expression in cell culture as well [66], suggesting that many more viruses have the potential to do so.

Expression of the HIV accessory protein Vif counteracts the viral restriction factor APOBEC3G and could result in abrogation of cellular HERV control [70] and *de novo *replication of HERV elements by reverse transcription. Restriction during reverse transcription by APOBEC3G was shown for HERV-K (HML-2) elements, both during *in vitro *replication as well as in the mutation pattern of ancient integrations [71,72].

## HERV proteins and antibodies in HIV infected patients

Increased expression of HERV-K Gag protein has been detected in both the CD4^+ ^and CD8^+ ^T-cell fractions from HIV-infected patients compared to T-cells from uninfected individuals [39]. It is remarkable that HERV-K Gag is also upregulated in CD8^+ ^T-cells that are normally not infected by HIV-1, which would suggest a more general and indirect mechanism of activation. No significant correlation was found between the HIV-1 pVL, CD4^+ ^or CD8^+ ^T-cell counts and HERV-K protein titers.

Antibodies to expressed HERV-K proteins are frequently found in several groups of patients, which is interesting because HERVs should be classified as self-antigens. Some studies did describe a HERV-K antibody response in HIV-1 infected persons, while others did not. No significant increase in serum antibodies against recombinant human teratocarcinoma-derived virus (HTDV)-HERV-K Env protein was seen in HIV-1 infected patients (15.2% versus 12.6% in healthy controls) [73]. Similar negative results were obtained by Vogetseder *et al*. [73] and Boller *et al*. [74], when examining the antibody response against HTDV/HERV-K Gag and Env proteins in HIV-1 positive individuals, and by Goedert *et al*. [75] in HIV-1 infected individuals with testicular cancer. In contrast, 70% of HIV-1 infected patients versus 3% of healthy controls tested positive for antibodies against HTDV/HERV-K Env in a fourth study [76]. A fifth study, employing HERV-K102 Env peptide serology reported similar numbers, with 70-80% of HIV-1 viraemic patients testing positive versus 2% of healthy controls and 18% of patients with herpes viraemia [43]. Interestingly, a patient seroconverting for anti-HIV-1 antibodies showed a parallel seroconversion for anti-HERV-K Env antibodies in the study by Vogetseder *et al*. [73]. Cross-reactivity is unlikely to explain this result, as the HERV-K and HIV-1 Env proteins have little amino acid similarity.

Testing urine specimens of HIV-1 infected individuals and healthy controls for antibodies against a peptide derived from the gammaretrovirus HERV-E Env protein showed that 36.4% and 81.3% of patients in the CDC AIDS categories A and B/C respectively, contained specific antibodies, while none of the HIV-negative controls tested positive [77]. Fourteen peptides derived from various endogenous retroviruses were used to search for IgM and IgG antibodies in HIV-1 infected and uninfected immunosuppressed persons by Lawoko *et al*. [78]. Three peptides, corresponding to the C-terminal half of the murine leukaemia virus capsid protein, a conserved domain of the HERV-H transmembrane domain, and part of HERV-K HML-3 encoded Pol, were found to bind IgG in both groups, with stronger binding of the latter two peptides in HIV-1 infected patients [78]. No correlation of either IgM or IgG binding with progression to AIDS was observed.

In conclusion, it is difficult to summarize these studies on anti-HERV antibodies in HIV-1 infected patients. Studies on HERV-K remain contradictory, while others investigated HERV families whose RNA expression levels during HIV infection have not been characterized.

## T-cell response to HERV in HIV infected patients

Although HERV proteins should be regarded as self-antigens, tolerance is often broken during neoplastic processes and also during HIV infection as demonstrated by a study that measured T-cell responses against HERV during HIV infection [41]. Peptides corresponding to HERV and HIV epitopes were tested for their ability to evoke a T-cell response *in vitro*. Ten peptides contained short regions of homology between HERV-H/-K/-L and HIV, respectively, and six others unique for HERV-L or HERV-W were used to estimate both putative cross-reactive and virus-specific T-cell responses. HERV-H and HERV-W cluster with the gammaretroviruses, and HERV-L has similarity to spumaviruses [79]. PBMC samples were collected from HIV-1 infected, HCV infected, and uninfected individuals. T-cell responses against HERV epitopes were detected only in HIV-1 positive patients, with no significant difference in reactions against unique HERV peptides versus HIV-related HERV peptides. This suggests that the response against HERV epitopes is distinct from the anti-HIV response. T-cell cross-reactivity was tested in three patients with a response to either the HIV-RT VL9 or HERV-L II9 peptide which share some amino acid similarity, but was found to be absent.

Anti-HERV T cell responses were broad and varied significantly among the study subjects [41]. The HERV-specific T-cell responses were inversely correlated with the HIV-1 pVL, raising the exciting possibility that these responses could be involved in the control of HIV replication. Reactivity to the unique HERV-L IQ10 peptide was found in 5/16 HIV-1 infected patients. Longitudinal analysis of three patients showed persistent responses to this peptide that declined in two patients when HAART was started. The third patient with the highest response to this peptide controlled HIV replication without therapy. HERV-specific CD8^+ ^T-cells were able to lyse cells presenting the corresponding peptide. If HIV-1 infected cells that present HERV peptides are subsequently lysed by HERV-specific CD8^+ ^T-cells, a decrease in HIV-1 pVL would be the result, explaining the observations in the three patients described above. In a follow-up study, anti-HERV T-cell responses were analysed in a larger cohort of untreated HIV-1 infected individuals [70]. The breadth and magnitude of the HERV response were again inversely correlated with HIV-1 pVL and positively associated with CD4^+ ^T-cell counts. The peptide that evoked the largest number of responses was derived from HERV-H Env. Other peptides tested originated from HERV-K (n = 14), HERV-L (n = 12), and HERV-W (n = 1).

An HIV-1 positive patient who controlled the infection for over 8 years showed HLA-B51 restricted responses to the HERV-K Pol epitope FAFTIPAI as well as to the corresponding HIV-1 epitope TAFTIPSI. The CD8^+ ^T-cells responding to the HERV epitope were found to be less activated and more differentiated than the cells targeting the HIV-1 epitope [70]. The cytomegalovirus-specific CD8^+ ^T-cell population in this patient did mimic the HERV response.

Overall, these results suggest that a robust CD8^+ ^T-cell response against the newly expressed HERV peptides presented by HIV infected cells may contribute to the control of HIV replication in blood. Indeed, a second study in vertically infected children showed a similar outcome: anti-HERV responses were inversely correlated with the HIV-1 plasma viral load and positively correlated with the CD4^+ ^T cell count [80]. Alternatively, Tandon *et al*. suggest that instead of a direct effect of anti-HERV responses, the correlations might be related to a more intact immune system in patients showing considerable anti-HERV activity.

## Anti-HERV vaccines as an approach to combat HIV?

T-cell responses that are effective in lowering the HIV-1 pVL and thus assist in controlling HIV replication are potential vaccine targets. In contrast to the replicating and rapidly mutating HIV-1 genome, HERVS are cellular genes that are not prone to mutation. Although HERV integrations are present in every cell in the body, they are expressed in a cell type and development specific way, making many HERV proteins rather specific antigens. Indeed, a HERV-K *env *transcript, HERV-K-MEL, encoded a melanoma-specific antigen that was recognized by cytolytic T lymphocytes [81]. Vaccination with peptides derived from another melanoma-specific antigen had been successful earlier in inducing tumour regression (see [81]), suggesting that vaccination with HERV-K-MEL peptides could be an effective strategy. A HERV-E transcript specifically expressed by renal cell carcinoma tissue and cell lines encoded a peptide that was recognized by CD8^+ ^T-cells after allogeneic hematopoietic stem cell transplantation [82]. Recognition resulted in tumour regression *in vivo*, an example of the therapeutic power of targeting HERV epitopes in malignancy.

The research performed by Garrison *et al*. [41], SenGupta *et al*. [70], and Tandon *et al*. [80] suggests that a similar approach is possible in HIV-1 infection. T-cell responses targeted at HERVs expressed by HIV-1 infected cells could be helpful in killing those cells. However, before embarking on the HERV approach, several basic issues need to be addressed. It is currently unclear which HERV epitopes are expressed by HIV-infected cells, at what magnitude and at what specificity. SenGupta *et al*. recorded CD8^+ ^T-cell responses against multiple HERV families in HIV-1 infected patients, including HERV-H, -K, -L, and -W [70]. HERV epitope recognition was found to vary among individuals, complicating the generation of a universal vaccine. It is also uncertain whether the discovered epitopes are expressed exclusively by HIV-1 infected cells, or whether there is also an indirect bystander effect on non-infected cells as reported by Contreras-Galindo *et al*. [39]. Furthermore, it is not clear if a similar pattern of HERV expression is apparent in all HIV-1 susceptible cell types e.g. in CD4^+ ^T-cells and monocytes. These issues need to be addressed *in vitro *and combined with the analysis of patient samples before HERV-vaccination approaches can be developed further.

## Conclusions

Infection of humans with the retrovirus HIV-1 has profound effects upon the resident, endogenous retroviruses. Transcripts and proteins representing diverse classes of endogenous retroviruses are upregulated. Direct interactions between HERVs and HIV-1 are limited as indicated by the lack of complementation, which probably relates to the low sequence similarity. Induction of certain HERV epitopes on HIV-1 infected cells renders these cells susceptible to lysis by CD8^+ ^T-cells, constituting a possible mechanism to control HIV-1 infection. It has been proposed to translate these observations into a vaccination approach with stable HERV-based antigens, but the detailed knowledge on the magnitude and specificity of HERV expression in HIV-1 infected cells is prerequisite for such applications.

## Competing interests

The author declares that she has no competing interests.

## Authors' contributions

ACvdK conceived the review topic and drafted the manuscript.
